# External Quality Assessment for Zika Virus Molecular Diagnostic Testing, Brazil

**DOI:** 10.3201/eid2405.171747

**Published:** 2018-05

**Authors:** Carlo Fischer, Celia Pedroso, Alfredo Mendrone, Ana Maria Bispo de Filippis, Antonio Carlos Rosário Vallinoto, Bergmann Morais Ribeiro, Edison Luiz Durigon, Ernesto T.A. Marques, Gubio S. Campos, Isabelle F.T. Viana, José Eduardo Levi, Luciano Cesar Scarpelli, Mauricio Lacerda Nogueira, Michele de Souza Bastos, Nathalia C. Santiago Souza, Ricardo Khouri, Sanny M. Costa Lira, Shirley Vasconcelos Komninakis, Cécile Baronti, Rémi N. Charrel, Beate M. Kümmerer, Christian Drosten, Carlos Brites, Xavier de Lamballerie, Matthias Niedrig, Eduardo Martins Netto, Jan Felix Drexler

**Affiliations:** German Centre for Infection Research, associated partner Charité–Universitätsmedizin, Berlin, Germany (C. Fischer, C. Drosten, J.F. Drexler);; LAPI, Hospital Universitário Professor Edgard Santos, Salvador, Brazil (C. Pedroso, C. Brites, E.M. Netto);; Fundação Pro-Sangue/Hemocentro de São Paulo, São Paulo, Brazil (A. Mendrone Jr., J.E. Levi);; Instituto Oswaldo Cruz, Rio de Janeiro, Brazil (A.M.B. de Filippis);; Federal University of Para, Belém, Brazil (A.C.R. Vallinoto);; University of Brasília, Brasília, Brazil (B. Morais Ribeiro);; University of São Paulo, São Paulo (E.L. Durigon, J.E. Levi, N.C.S. Souza);; Oswaldo Cruz Foundation, Pernambuco, Brazil (E.T.A. Marques Jr., I.F.T. Viana);; Universidade Federal da Bahia, Salvador (G.S. Campos);; Diagnósticos da América—DASA, São Paulo (J.E. Levi, L.C. Scarpelli);; Hospital Israelita Albert Einstein, São Paulo (J.E. Levi, S.M.C. Lira);; Faculdade de Medicina de São José do Rio Preto, São José do Rio Preto, Brazil (M.L. Nogueira);; Fundação de Medicina Tropical Dr. Heitor Vieira Dourado, Manaus, Brazil (M. de Souza Bastos);; Fundação Oswaldo Cruz, Salvador, Brazil (R. Khouri);; Federal University of São Paulo, São Paulo (S.V. Komninakis);; Aix Marseille Université, Marseille, France (C. Baronti, R.N. Charrel, X. de Lamballerie);; Assistance Publique-Hopitaux Marseille, Marseille (C. Baronti, R.N. Charrel, X. de Lamballerie);; University of Bonn Medical Centre, Bonn, Germany (B.M. Kümmerer);; Robert Koch Institute, Berlin, Germany (M. Niedrig)

**Keywords:** Zika virus, viruses, diagnostics, surveillance, real-time RT-PCR, vector-borne infections, Americas, Brazil

## Abstract

We conducted an external quality assessment of Zika virus molecular diagnostic tests in Brazil using a new Zika virus standard. Of 15 laboratories, 73% showed limited sensitivity and specificity. Viral load estimates varied significantly. Continuous quality assurance is required for adequate estimates of Zika virus–associated disease and determination of patient care.

The catastrophic Zika virus outbreak in the Americas has affected millions of persons. Brazil was the most affected country and reported ≈95% of all cases of suspected Zika virus–associated congenital disease (*1*). Limited sensitivity and specificity of tests hampers serologic detection of Zika virus–specific antibodies in tropical regions (*2*). Thus, real-time reverse transcription PCR (RT-PCR) has been key for diagnosing acute Zika virus infection and for use in epidemiologic studies (*3*–*5*). However, Zika virus molecular diagnostic testing is challenged by short-term viremia and low viral loads (*3*).

A recent external quality assessment (EQA) in Europe revealed that 60% of laboratories need to improve molecular Zika virus detection (*6*). Laboratories in affluent countries conduct Zika virus diagnostic testing predominantly in travelers returning from tropical regions. In resource-limited settings to which multiple co-circulating arboviruses are endemic, the diagnostic demands differ entirely. To evaluate the diagnostic landscape in the region most affected by Zika virus, we performed an EQA of molecular Zika virus diagnostic testing in Brazil during 2017.

## The Study

Fifteen laboratories from 7 Brazilian states participated in this study; these laboratories are spread across ≈2,500 km longitude, including the areas most affected during Brazil’s Zika virus outbreak (*1*). Participants were university laboratories, hospital laboratories, federal research institutes supporting public health services, and a diagnostic testing company. We provided EQA panels to all laboratories. Each panel comprised 12 lyophilized samples containing inactivated full virus spiked into human plasma tested negative for arboviruses beforehand. The panel consisted of 4 Zika virus–positive specimens of 10^3^–10^6^ RNA copies/mL to assess sensitivity and determine viral load. Zika virus–negative specimens to assess specificity comprised dengue virus serotypes 2 and 4, Japanese encephalitis virus, St. Louis encephalitis virus, West Nile virus, yellow fever virus, and chikungunya virus at ≈10^5^ 50% tissue culture infective dose/mL each and a negative plasma specimen (Table 1). Moreover, each panel included the international World Health Organization (WHO) Zika virus standard for quantification (*7*). However, the WHO standard has limited availability. Importation of the WHO standard may be restricted by countries that perceive heat-inactivated materials that derive from live virus as potentially infectious. Therefore, we designed and acquired a Zika virus armored RNA (Asuragen, Austin, TX, USA). The Zika virus armored RNA is a synthetic RNA covering the target sites of 9 Zika virus–specific real-time RT-PCRs as described previously (*3*), encapsulated into bacteriophage proteins. This highly stable, noninfectious, pseudoviral particle can be used as a universal control for the covered assays, shipped without biosafety concerns, and used as a control for both nucleic acid preparation and RT-PCR. 

**Table 1:** External quality assessment of 15 laboratories from 7 states of molecular diagnostic testing for Zika virus, Brazil*

Lab ID	Zika virus, copies/mL^3^				CHIKV	DENV-2	DENV-4	JEV	SLEV	WNV	YFV	Plasma	Correct result/no. tested
	MRS, 8.1 × 10^5^	MRS, 7.0 × 10^3^	MRS, 1.3 × 10^3^	MR766, 2.1 × 10^3^									
	S-7	S-4	S-12	S-9	S-10	S-5	S-8	S-2	S-11	S-6	S-3	S-1	
3	+	+	+	+	–	–	–	–	–	–	–	–	12/12
11	+	+	+	+	–	–	–	–	–	–	–	–	12/12
12	+	+	+	+	–	–	–	–	–	–	–	–	12/12
13	+	+	+	+	–	–	–	–	–	–	–	–	12/12
1	+	+	+	(–)	–	–	–		–	–	–	–	11/12†
6	+	+	+	(–)	–	–	–	–	–	–	–	–	11/12
10	+	+	(–)	+	–	–	–	–	–	–	–	–	11/12
4	+	+	(–)	(–)	–	–	–	–	–	–	–	–	10/12
7	+	+	(–)	(–)	–	–	–	–	–	–	–	–	10/12
9	+	+	+	+	–	–	(+)	–	–	(+)	(+)	–	9/12
2	+	+	(–)	(–)	(+)	–	–	(+)	–	–	–	–	8/12
14	+	+	(–)	+	(+)	–	–	–	–	(+)	(+)	(+)	7/12
15	+	(–)	(–)	(–)	–	(+)	–	(+)	(+)	–	–	–	6/12
5	+	+	+	+	(+)	(+)	(+)	(+)	(+)	(+)	(+)	(+)	4/12
8	+	+	NT	+	NT	(+)	(+)	(+)	NT	(+)	(+)	(+)	3/9
Total‡	15/15 (100)	14/15 (93)	8/14 (57)	9/15 (60)	11/14 (79)	12/15 (80)	12/15 (80)	11/15 (73)	12/14 (86)	11/15 (73)	11/15 (73)	12/15 (80)	Average 9.2/11.8

*Positive samples contained different amounts of Zika virus strain MRS_OPY_Martinique_PaRi_2015 (representing the Asian lineage, including the outbreak strain in the Americas) or Zika virus strain MR766 (representing the African lineage). Zika virus–negative controls contained human plasma, CHIKV, DENV seroypes 2 and 4, JEV, SLEV, WNV, or YFV. Samples were prepared from 0.2 mL phosphate buffered saline supplemented with 20% human plasma and spiked with virus culture supernatants. Viruses were heat inactivated before lyophilization. Human plasma was tested negative for viral RNA and for real-time reverse transcription PCR (RT-PCR) inhibition before spiking of viral cell culture supernatants. Detection of different samples was analyzed by the exact test of goodness-of-fit with p>0.1 being significant. The parameter value defining the expected ratio of correct tests was set to 0.99. Only the 2 samples containing the highest Zika virus loads were tested correctly at statistical significance (p = 1.0 and p = 0.134, respectively). Detection of all other samples showed p values of <0.009. All laboratories except 1 used an assay published by Lanciotti et al. (2). CHIKV, chikungunya virus; DENV, dengue virus; ID, identification number; JEV, Japanese encephalitis virus; NT, samples not tested; S, sample no.; SLEV, St. Louis encephalitis virus; WNV, West Nile virus; YFV, yellow fever virus; +, correct positive result; –, correct negative result; (+), false-positive; (–), false-negative. †This laboratory used the RealStar Zika Virus RT-PCR Kit (Altona Diagnostics, Hamburg, Germany). ‡Correct results/total results (%).

We asked all laboratories to conduct molecular Zika virus diagnostics as routinely done with clinical samples and to quantify Zika virus–positive specimens using both standards. All but 1 laboratory used the same real-time RT-PCR protocol developed by Lanciotti et al. (*2*), highlighting the wide dissemination of this assay in Brazil and suggesting comparability of test results within this study (Table 1). We found no significant difference between samples containing comparable quantities of the Asian and the African Zika virus lineage, suggesting suitability of the protocols for both lineages (p = 0.313 by Fisher exact test).

**Figure 1:**
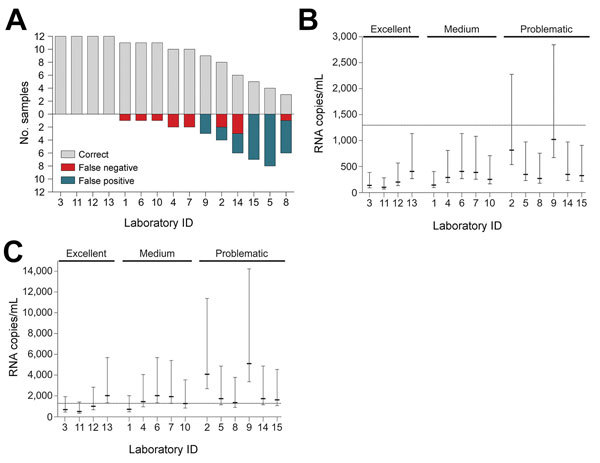
External quality assessment (EQA) performance and lower limits of detection (LODs) for Zika virus molecular diagnostic testing, Brazil. A) EQA performance of individual laboratories. Gray bars above the baseline indicate correctly tested samples; bars below the baseline indicate incorrectly tested samples. Laboratories are sorted by the quantity of correct samples and the numeric order of the laboratory identification numbers. Laboratory 8 tested only 9 of 12 samples. B) Projected 95% LODs of participating laboratories under optimal conditions; C) projected 95% LODs of participating laboratories assuming a 5-fold loss in sensitivity. LODs were projected using the technical LOD of the Lanciotti et al. assay as analyzed previously (*2*), input and elution volumes, and real-time reverse transcription-PCR setups. Efficacy of RNA extraction was assumed to be 100%. Whiskers indicate 95% CIs. Dotted line indicates the lowest Zika virus RNA titer of an EQA specimen. Laboratories are grouped according to their EQA performance as excellent, medium, or problematic. LODs did not differ significantly among groups (p>0.05 by Kruskal-Wallis test)..

EQA results varied among laboratories. Of 15 laboratories, 4 (27%) reported correct results for all samples. Five (33%) reported 1 or 2 false-negative results from samples with low Zika virus concentrations (Table 1; Figure 1, panel A). EQA participants correctly tested only the 2 samples containing the highest Zika virus concentrations of 8.1 × 10^5^ and 7.0 × 10^3^ copies/mL (exact test of goodness-of-fit p = 1.00 and p = 0.14, respectively). This finding suggests a potential lack of sensitivity that may be problematic given that viral loads of 10^3^–10^4^ copies/mL are commonly observed in Zika virus–infected patients (*3*).

Six (40%) laboratories reported >3 false results, including at least 2 false-positive detections of Zika virus–negative specimens. No heterologous flavivirus was particularly affected by false-positive detection, suggesting that false-positive results did not result from unspecific binding of assay oligonucleotides (Table 1). Instead, false-positive results hint at the possibility of laboratory contamination potentially resulting from virus isolation attempts or PCR amplicons generated during prior Zika virus experimentation.

EQA performance varied according to the way viral RNA was prepared. The 8 laboratories conducting Zika virus detection using automated platforms performed generally superior (n = 8; Youden index, 0.661) compared with the 7 laboratories conducting manual RNA extraction (Youden index, 0.446) (Table 2). This finding might indicate an increased risk for contamination during manual RNA preparation. However, automated RNA preparation also might represent a proxy for more affluent settings of those laboratories.

**Table 2:** Viral RNA preparation of individual laboratories in an external quality assessment for Zika virus molecular diagnostic testing, Brazil

Lab ID	Extraction method	Extraction kit	Input volume, μL	Elution volume, μL	PCR template volume, μL
1	Manual	QIAamp Viral RNA Mini Kit (QIAGEN, São Paulo, Brazil)	140	50	10
2	Manual	QIAamp Viral RNA Mini Kit (QIAGEN)	200	200	5
3	Automated	QIAsymphony DSP Virus/Pathogen Midi Kit (QIAGEN)	200†	60	8.8
4	Automated	Maxwell 16 Viral Total Nucleic Acid Purification Kit (Promega, São Paulo, Brazil)	140	50	5
5	Manual	QIAamp Viral RNA Mini Kit (QIAGEN)	140	60	5
6	Automated	QIAsymphony DSP Virus/Pathogen Kit (QIAGEN)	200	100	5
7	Manual	QIAamp Viral RNA Mini Kit (QIAGEN)	140	60	4.5
8	Automated	Maxwell 16 Viral Total Nucleic Acid Purification Kit (Promega)	150	50	5
9	Manual	High Pure Viral Nucleic Acid Kit (Roche, São Paulo, Brazil)	200	50	1
10	Manual	QIAamp Viral RNA Mini Kit (QIAGEN)	160	50	5
11	Automated	NucliSENS easyMAG Kit (bioMérieux, Rio de Janeiro, Brazil)	200†	50	10
12	Automated	Magna Pure Compact Nucleic Acid Isolation Kit I—Large Volume (Roche)	200‡	50	5
13	Automated	Maxwell 16 Viral Total Nucleic Acid Purification Kit (Promega)	100	50	5
14	Manual	QIAamp Viral RNA Mini Kit (QIAGEN)	140	60	5
15	Automated	Abbot mSample Preparation System RNA (4 × 24 prep) (Abbott, São Paulo, Brazil)	200	80	5

*All details are listed as declared by the participants. ID, identification. †Laboratory that filled the 200 µL provided in this external quality assessment panel to higher standard extraction input volumes ranging from 500 µL to 1,200 µL using human plasma tested negative for arboviral infections beforehand. ‡Laboratory that filled the 200 µL provided in this external quality assessment panel to higher standard extraction input volumes ranging from 500 µL to 1,200 µL using sterile nuclease-free water.

As previously reported (*3*), RNA extraction critically influences the clinical lower limit of detection (LOD). Although all participants used highly sensitive real-time RT-PCRs, clinical LODs varied considerably because of different RNA extraction protocols (Figure 1, panel B). Lack of detection of low-concentration EQA samples is thus not surprising because even a small decrease in sensitivity readily causes clinical LODs above the concentration of the lowest EQA panel specimen (Figure 1, panel C). This finding highlights that optimized RNA extraction protocols are crucial for sensitive Zika virus diagnostics.

**Figure 2:**
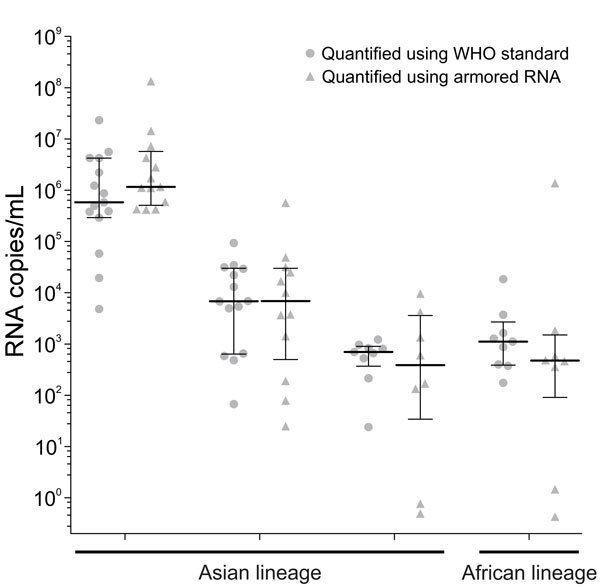
Quantification of Zika virus–positive samples using WHO Zika virus and armored RNA testing standards, Brazil. Zika virus–positive samples contained either inactivated strain MRS_OPY_Martinique_PaRi 2015 (Asian lineage) or strain MR766 (African lineage). Horizontal lines indicate median of the calculated Zika virus; whiskers indicate interquartile ranges. Statistical analysis was performed using GraphPad Prism 5.03 (GraphPad Software, Inc., La Jolla, USA). WHO, World Health Organization.

Quantification of Zika virus loads did not differ significantly between use of the armored RNA and the WHO Zika virus standard, with only 0.76 log_10_ median deviation between results (p = 0.429 by Wilcoxon signed rank test). This observation suggests usability of the armored RNA for Zika virus quantification in tropical regions. Irrespective of the standard, viral load determinations among laboratories were comparable with 0.12–0.88 log_10_ median deviations of viral load estimates among laboratories for individual Zika virus specimens. However, we also observed drastic deviations of up to 6 orders of magnitude (Figure 2), suggesting that caution must be taken upon comparing viral load determinations as markers for severe Zika virus disease (*8*,*9*) among different laboratories.

## Conclusions

Some laboratories in Brazil showed suboptimal sensitivity and specificity of Zika virus diagnostic testing. However, these laboratories performed comparably to those in Europe (*6*). Neither sensitivity nor specificity differed significantly between laboratories in Brazil compared with those in Europe (p = 0.767 and p = 0.324, respectively, by Fisher exact test). Similarly, the proportion of perfectly performing laboratories in this EQA (27%) was comparable with previous EQAs of flavivirus molecular diagnostics, including yellow fever virus (18%), dengue virus (24%), and West Nile virus (27%) (*10*–*12*). Flavivirus molecular diagnostics are thus generally challenging and benefit greatly from controls, such as those provided in this EQA. This study underscores the need to combine RT-PCR and serologic testing in Zika virus diagnostic testing, despite their inherent limitations (*3*).

Independently of the challenges of Zika virus molecular detection, because of taxation and distributor margins, RT-PCR reagents in Latin America are usually 100%–200% more expensive than in affluent countries (*13*). Limited resources and relatively higher costs potentially force laboratories in Brazil to seek inferior, more affordable solutions. Enhanced access of laboratories in tropical regions to state-of-the-art reagents is thus an unresolved key component of outbreak response. Further EQAs in Brazil should involve state laboratories that carry a large proportion of Zika virus testing within the public health care system. Unfortunately, the state laboratories we contacted for this EQA could not participate because of limited resources.

Finally, lack of sensitivity directly affects estimates of the absolute risk for Zika virus–induced congenital disease upon maternal infection during pregnancy (*14*). False-positive results potentially have dramatic consequences for patients, as illustrated by a >90% increase in illegal abortion requests in Latin America during the 2016 Zika virus epidemic (*15*). Our results emphasize the need for continuous quality assessments of Zika virus diagnostic testing globally.
